# Nobody’s Perfect: Choice of the Buffer and the Rate of Cu^2+^ Ion–Peptide Interaction

**DOI:** 10.1021/acs.inorgchem.4c01797

**Published:** 2024-06-14

**Authors:** Radosław Kotuniak, Dobromiła Z. Sudzik, Iwona M. Ufnalska, Wojciech Bal

**Affiliations:** Institute of Biochemistry and Biophysics, Polish Academy of Sciences, Pawińskiego 5a, 02-106 Warsaw, Poland

## Abstract

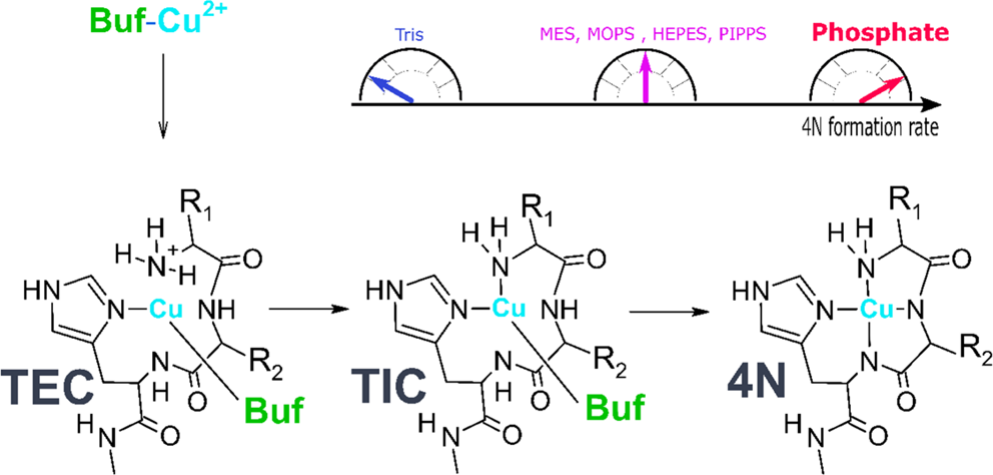

The choice of correct pH buffer is crucial in chemical studies modeling biological processes involving Cu^2+^ ions. Popular buffers for physiological pH are known to form Cu(II) complexes, but their impact on kinetics of Cu(II) complexation has not been considered. We performed a stopped-flow kinetic study of Cu^2+^ ion interactions with four popular buffers (phosphate, Tris, HEPES, and MOPS) and two buffers considered as nonbinding (MES and PIPPS). Next, we studied their effects on the rate of Cu^2+^ reaction with Gly-Gly-His (GGH), a tripeptide modeling physiological Cu(II) sites, which we studied previously at conditions presumably excluding the buffer interference [KotuniakR.; Angew. Chem., Int. Ed.2020, 59, 11234–1123910.1002/anie.202004264PMC738391232267054]. We observed that (i) all tested pH 7.4 buffers formed Cu(II) complexes within the stopped-flow instrument dead time; (ii) Cu(II)-peptide complexes were formed via ternary complexes with the buffers; (iii) nevertheless, Good buffers affected the observed rate of Cu(II)-GGH complex formation only slightly; (iv) Tris was a competitive inhibitor of Cu(II)-GGH complexation; while (v) phosphate was a reaction catalyst. This is particularly important as phosphate is a biological buffer.

## Abstract 2

Stopped-flow kinetic studies of Cu^2+^ reaction with GGH, a tripeptide modeling biological Cu(II) sites, revealed that popular buffers phosphate, Tris and Good buffers HEPES, MOPS, MES, and PIPPS affected the observed formation rate of the final 4N complex via ternary complexes with reaction intermediates. Good buffers had only a slight effect, Tris was a competitive inhibitor, and biologically relevant phosphate was a strong catalyst. These results highlight the impact of buffer choice in bioinorganic studies.

## Introduction

Biological processes, including those involving Cu^2+^ ions, take place in pH-buffered environments necessary for the smooth running of (bio)chemical reactions. Unfortunately, physiological buffers cannot be fully employed in quantitative laboratory studies aimed to reproduce and understand such reactions because they introduce too much physical and chemical interference to the studied systems. For example, the blood serum buffer is composed of bicarbonate/carbonic acid, phosphates, and serum albumin protein.^1^ The bicarbonate buffer is instable in vitro, and serum albumin forms very strong Cu(II) complexes.^2^ Therefore, it is necessary to use an alternative “chemical” buffer. This, however, is not an easy task. Known buffers with a satisfactory capacity in the physiological pH range, roughly 7.0–7.7, such as phosphate, HEPES (2-[4-(2-hydroxyethyl)piperazin-1-yl]ethanesulfonic acid), Tris (2-amino-2-hydroxymethylpropane-1,3-diol), ACES (*N*-(2-acetamido)-2-aminoethanesulfonic acid), or MOPS (3-morpholinopropane-1-sulfonic acid), form Cu(II) complexes with binding constants in the range of 10^3^ M^–1^ or higher.^3−7^ According to a comprehensive review by Ferreira et al.,^8^ most of the buffers listed as “noncoordinating” in 1966 by Good and co-workers,^9^ and updated by Rorabacher and co-workers in 1997,^10^ actually form Cu(II) complexes. Summarizing the data reviewed by Ferreira et al., only MES (2-(*N*-morpholino)ethanesulfonic acid) is noncoordinating in its buffering range around pH 6. A few buffers cited by Ferreira et al. have not yet been rigorously studied regarding Cu(II) complexation. For example, PIPPS ((1,4-piperazinedipropanesulfonic acid)), which has a p*K*_a_ of 7.97 and provides effective buffering in the pH range of 7–9,^11^ was used in a study of dissolution of cuprous minerals, with no coordination-based effects reported.^12^

Parallel interactions of buffers with Cu^2+^ ions are known to affect the primary reaction equilibria with the ligands of interest. If not taken into account, these interactions may result in significant errors of stability constant determinations^13^ and can even change the course of reaction by forming ternary complexes.^14^ Very little is known, however, about the effects that buffers may exert on the kinetics of such reactions.

The ATCUN/NTS motifs consist of N-terminal tripeptide sequences, characterized by the free N-terminus, a His residue in position three and any intervening amino acid, except Pro.^15,16^ These motifs bind Cu(II) ions spontaneously in a broad pH range, yielding square-planar four-nitrogen (4N) complexes characterized with high stability (log ^C^*K* = 12–15 at pH 7.4). They are also inert in reactions of Cu(II) ion dissociation/exchange.^17^ The ATCUN/NTS motifs are present in many extra- and intracellular human proteins, but in most cases, their biological function remains to be established.^18^ Notably, these motifs are present in human serum albumin (Asp-Ala-His N-terminal sequence) and human Ctr1 cell membrane copper transporter (Met-Asp-His N-terminal sequence), two proteins involved in copper transport.^13^ ATCUN/NTS peptides have been massively used in chemical studies modeling the chemical reactivity of their parent proteins with respect to Cu(II) ions.^16^

**Scheme 1:**
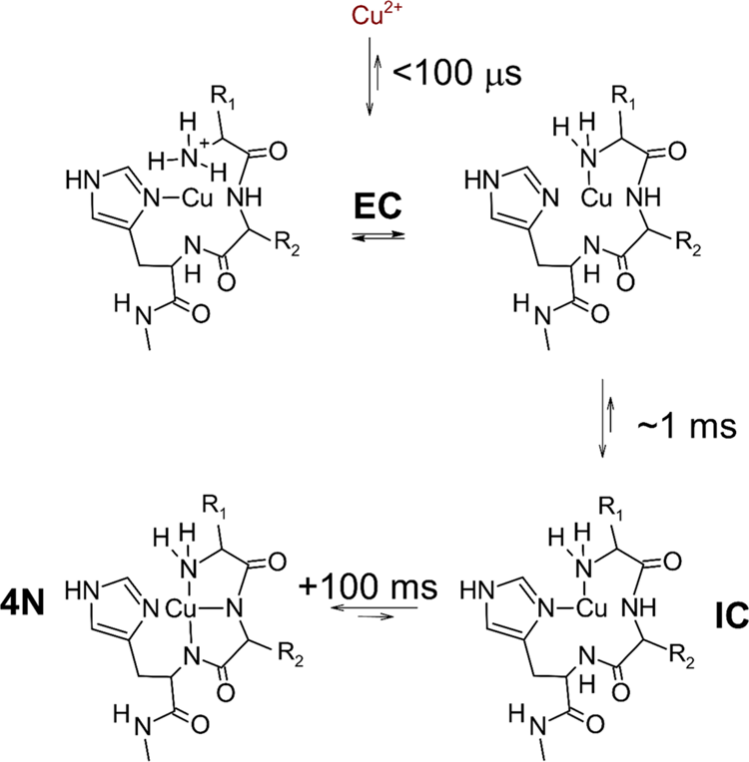
Course of Cu^2+^ Ion Reaction with ATCUN/NTS Peptides^19−23^ The structures of the Early Complex (EC) and Intermediate Complex (IC) were inferred from their spectroscopic properties, while the structure of the final 4N complex is well-established in the literature. Typical half-times for individual reaction steps are indicated. IC represents a number of interconverting conformers.

Recently, we and our collaborators used fast kinetic methods (stopped-flow and freeze-quench) coupled with EPR and ultraviolet–visible (UV–vis) spectroscopies to elucidate the molecular mechanism of Cu^2+^ complexation by Gly-Gly-His (GGH), the simplest ATCUN/NTS peptide model.^19,20^ In a separate study, this Cu(II) binding kinetics was also studied for the N-terminal peptide of Alzheimer’s Disease-associated peptide Aβ_4–16_.^21^ The reaction consists of three major steps, presented in Scheme 1, which are characterized by distinct coordination environments for the Cu(II) ion. In the initial one, completed within 100 μs, the Cu^2+^ aqua ion forms a single bond with the peptide nitrogen, with a preference for imidazole ring atoms (1N complex, Early Complex (EC) in Scheme 1). The EC was documented by EPR spectra of freeze-quenched samples. The next species (2N complex, IC in Scheme 1) is formed within 1 ms and contains a macrochelate loop complemented by the other terminal nitrogen donor (Gly amine if the initial anchoring was at the imidazole). This species is more stable and was directly detected in stopped-flow experiments. It eventually undergoes a rearrangement into a thermodynamically stable 4N complex (*t*_1/2_ ∼ 100 ms) in which the equatorial Cu(II) coordination sphere is fully saturated in a square-planar structure. Additional interactions in the first and second coordination sphere of the IC enabled by amino acid substitutions in positions upstream or directly downstream of the His-3 residue can increase the lifetime of the IC substantially (up to several seconds) but do not affect the overall course of the reaction.^22^ We recently explored conceptually the impact of this reaction mechanism on the biological copper transport processes, noting the correspondence of lifetimes of the intermediate species with physiological cycles.^23^

The EC and IC contain the Cu(II) ion bound to just one or two donor atoms (1N and 2N, respectively; Scheme 1). This makes them prone to ternary interactions by exchanging the labile water molecules present at other positions around the Cu(II) ion in *D*_4*h*_ symmetry. Such interaction was observed for HEPES buffer. It affected the rate of Cu^2+^ ion binding to the Aβ_4–16_ peptide.^21^ Inspired by this observation, here we applied stopped-flow kinetics to investigate how buffers suitable for pH 7.4 affect the Cu^2+^ reaction with GGH. We considered HEPES, Tris, MOPS, and phosphate, which are commonly used in biochemical studies, and PIPPS as a candidate weakly interacting buffer. For the sake of consistency, we also performed experiments with MES, as in the previous GGH study.^19^ The MES p*K*_a_ is 6.15, so it could not be used to buffer the pH 7.4.^24^

**Scheme 2:**
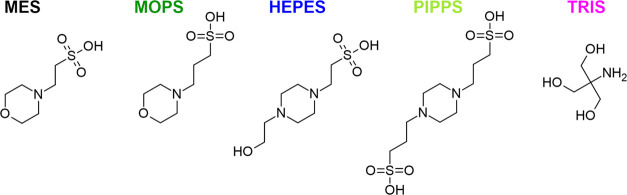
Structures and Acronyms of Organic Buffers Used in This Study The molecules are shown in their formally neutral forms. The colors of acronyms correspond to the color code adopted to present experimental data throughout the article.

Structures and systematic names of the studied buffers are listed in Scheme 2. From the structural point of view, MES and MOPS are morpholine derivatives while HEPES and PIPPS contain the piperazine ring. Tris is the main representative of its own buffer family containing the characteristic primary amine, and phosphate is the main component of physiological buffer, most commonly used as phosphate-buffered saline (PBS), the common buffering medium in biochemistry and cell biology.

## Materials and Methods

GGH, CuCl_2_·2H_2_O, HEPES, PIPPS, MOPS, MES, and Tris were purchased from Merck (Darmstadt, DE). HCl, NaOH, and disodium and monosodium phosphates were purchased from ChemPur (Piekary Śląskie, PL).

Reactions of Cu^2+^ ions with buffers were performed using 3.2 mM CuCl_2_ solution, acidified with HCl at pH 4 to prevent the Cu(OH)_2_ precipitation prior to the reaction, and 400 mM solutions of phosphate, HEPES, PIPPS, MOPS, and Tris, pH 7.4, and MES, pH 6.0. The choice of a 125-fold excess of buffer over the Cu^2+^ ions was dictated by test experiments, where a significant pH drift of the reaction mixture was noticed at a lower buffer excess. The same buffer excess was used in our previous studies.^19,22^ The CuCl_2_ solutions were mixed with buffer solutions at 1:1 ratios in an SFM-300 diode-array stopped-flow apparatus (BioLogic, Seyssinet-Pariset, France) with the 400–900 nm detection range in a 1 cm quartz cuvette. The dead time of the stopped-flow instrument was 2 ms. The experiments were carried out at 25 °C, with a typical flow rate of 15 mL/min. After each series of measurements, the system was rinsed 3 times with 0.2% HCl and 3 times with water after each run in order to remove traces of Cu(OH)_2_ precipitates. The course of reaction was monitored in 2 time windows: 1.5 and 300 s. In both cases, the number of collected spectra was 1000. In short runs, the time interval between the spectra was 1.5 ms (thus providing the first spectrum at 3.5 ms). In long runs, the integration time was increased to 32 ms (first spectrum at 34 ms); the first 500 spectra were recorded with 32 ms intervals and the remaining 500 spectra with 576 ms intervals.

Reactions of Cu^2+^ ions with the GGH peptide in the presence of buffers were performed similarly, with 4 mM GGH dissolved in respective 400 mM buffers mixed at a 1:1 volume ratio, using initial Cu^2+^ concentrations between 1.4 and 3.6 mM (final concentrations: 2 mM GGH in 200 mM buffer, 0.7–1.8 mM Cu^2+^). The reactions were monitored for 1.5 s with a time resolution of 1.5 ms or for 10 s with a time resolution of 10 ms.

The phosphate concentration dependence experiments were performed using initial concentrations of 1.6 mM Cu^2+^ and 2.0 mM GGH in phosphate buffers, pH 7.4, of various concentrations between 50 mM and 1 M (final concentrations: 0.8 mM Cu^2+^ and 1 mM GGH in 25–500 mM phosphate buffer).

The pH dependence of reaction was studied for MES, phosphate, HEPES, and MOPS buffers in the pH ranges of 5.5–6.5, 6.5–7.7, 6.4–7.7, and 6.4–7.8, respectively, using initial concentrations of 3.2 mM Cu^2+^ and 4.0 mM GGH in 400 mM buffer (final concentrations: 1.6 mM Cu^2+^ and 2.0 mM GGH in 200 mM buffer). The reactions were monitored for 1.5 s with a time resolution of 1.5 ms.

All reactions were repeated at least 5 times under each experimental condition. The kinetic data processing and the analyses of concentration and pH dependences of rate constants were performed by using Origin2024. The observed rate constants (*k*_obs_) were determined by fitting the absorption values at 525 nm to the monoexponential function, unless indicated otherwise. The UV–vis spectra were smoothed, when necessary, using a Fast Fourier Transform (FFT) smoothing procedure implemented in Origin2024, using a 45-point filter window.

## Results and Discussion

### Interactions of Cu^2+^ Ions with Buffers in the Absence of GGH

Stopped-flow experiments, aiming at investigating early stages of Cu^2+^ ions interactions with buffers, were preceded by benchtop tests consisting of manual mixing of appropriate reactants according to the protocol further implemented in stopped-flow studies: 3.2 mM CuCl_2_ + 400 mM buffer, equal volumes, pH 6.0 for MES, pH 7.4 for other buffers. For the sake of comparison, two additional reactions of Cu^2+^ ions were performed, one with an equal volume of 50 mM NaOH and another with an equal volume of distilled water. The samples were left for 60 min on the bench and checked visually. Tris buffer and pure water were the only ones that did not exhibit formation of Cu(II) precipitates.

**Figure 1:**
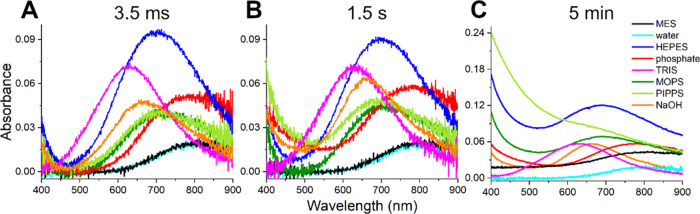
Absorption spectra resulting from mixing of 3.2 mM Cu(H_2_O)_6_^2+^ ions (delivered by dissolving CuCl_2_ in distilled water) with the same volumes of 400 mM buffers, pH 6.0 for MES and pH 7.4 for other buffers (final concentrations: 1.6 mM Cu(II) and 200 mM buffer). Additional reactions for 3.2 mM Cu(H_2_O)_6_^2+^ ions with water (final pH 4.3) and with 50 mM NaOH (final pH 12.4) were also performed. Panels A–C present absorption spectra at different time points of the reaction: (A) 3.5 ms (initial spectra), (B) 1.5 s, and (C) 5 min (end of observation period). The spectra are color-coded according to the labels in the graph throughout the article.

Next, these reactions were studied on a stopped-flow instrument equipped with a diode-array detector. The data were collected with two different time intervals, enabling monitoring the reaction in two distinct time windows: from 3.5 ms to 1.5 s and from 34 ms to 5 min. The complete sets of data are provided in Figures S1 and S2, whereas comparisons of initial (at 3.5 ms) and final (separately at 1.5 s and 5 min) absorption spectra are presented in Figure 1. The shorter of these observation periods corresponded to the typical time window of GGH reactions in MES buffer, studied previously.^19^ The longer one was considered as sufficiently corresponding to macroscopic observation times and still assuring the stability of the instrument baseline. Figure S3 presents the kinetic traces recorded at the d–d band maximum over the 1.5 s observation period. These traces clearly confirm that the binding of buffer molecules to the Cu^2+^ ion occurred within the dead time of the measurements.

At 3.5 ms, the spectrum of Cu^2+^/MES, centered at 812 nm, was identical to that of CuCl_2_ proving no interaction with buffer molecules under examined conditions (Figure 1A, black and light blue lines). Unlike MES, for all other buffers, a significant increase of the d–d band intensity, accompanied by a blue shift, was observed. Such a clear spectral change vs that of the Cu(H_2_O)_6_^2+^ ion indicates the formation of Cu(II)/buffer complexes. The fast rates of these reactions are not surprising. In our previous study of Cu(H_2_O)_6_^2+^ ion reaction with GGH, we observed the completion of formation of the first peptide complex (EC, Scheme 1) within 100 μs.^19^ With buffer concentrations 2 orders of magnitude higher than GGH, we can estimate the timespan of this second-order reaction to be within single microseconds.

The parameters of these initial spectra are presented in Table 1 and compared with the available literature values. Those for Cu(H_2_O)_6_^2+^ (in MES, pH 6.0), the transiently soluble Cu(OH)_2_, the CuHPO_4_ species, and the steady state Tris complexes are in agreement with previous publications.^3,6,25,26^ The HEPES complex is not stable at pH 7.4, slowly decomposing into a hydroxide species, but its coordination mode was extrapolated from pH and spectroscopic titrations.^6^ The d–d band shapes and absorption maxima for MOPS and PIPPS are very similar to each other and red-shifted by just 10 nm compared to HEPES. These spectra were not published before. They indicate a coordination mode like in HEPES, with one nitrogen atom in the Cu(II) coordination sphere.

**Table 1:** Parameters and Assignments of the d–d Bands Recorded at the Beginning (3.5 ms) and End (300 s) of Cu(H_2_O)_6_^2+^ Ion Reactions with the Studied Buffers^a^

	at 3.5 ms		at 300 s		
buffer^b^	λ_max_ (nm)	ε (M^–1^ cm^–1^)	λ_max_ (nm)	initial coordination mode	refs
NaOH (pH 12.4)	668	29	665	Cu(OH)_2_/Cu(OH)_4_^2–^	(25,26)
phosphate	786	31	779	HPO_4_^2–^	(3)
HEPES	701	59	690	N + OH^–^	(4)
MOPS	715	25	691	N + OH^–^	this work^c^
PIPPS	712	24	d	N + OH^–^	this work^c^
Tris	631	54	636	N+ RO^–^/2N+ RO^–^	(6)
MES (pH 6.0)	812	12	807	aqua ion	this work
water (pH 4.0)	812	12	812	aqua ion	this work

a The reaction with sodium hydroxide is included for comparison.

b pH 7.4, Unless stated otherwise.

c Coordination mode inferred by analogy with HEPES.

d Values could not be established.

**Figure 2:**
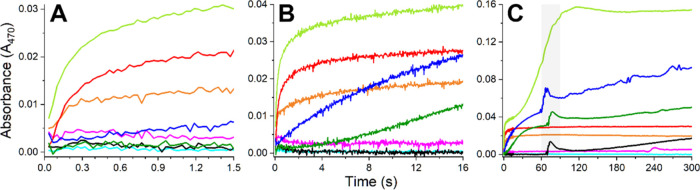
Absorbance changes at 470 nm corresponding to Cu(II) precipitates formation, resulting from mixing of 3.2 mM Cu(H_2_O)_6_^2+^ ions (delivered by dissolving CuCl_2_ in distilled water) with the same volumes of 400 mM buffers, pH 6.0 for MES and pH 7.4 for other buffers (final concentrations: 1.6 mM Cu(II) and 200 mM buffer). Additional reactions for 3.2 mM Cu(H_2_O)_6_^2+^ ions with water and 50 mM NaOH (final pH of 12.4) were performed. Panels (A–C) present kinetic traces in different reaction time windows: (A) 3.5 ms–1.5 s, (B) 34 ms–16 s, (C) 34 ms–300 s. In panel (C), 60–90 s time window was marked in gray. Kinetic traces are color-coded according to the labels in Figure 1: red, phosphate; orange, NaOH; black, MES; dark blue, HEPES; light blue, water; dark green, MOPS; light green, PIPPS; magenta, TRIS.

Quite remarkably, not only Tris but also several other studied systems remained well-soluble within 1.5 s after mixing the reagents. The solutions of MOPS and MES exhibited no trace of turbidity, HEPES exhibited it just a little, while the hydroxide solution, PIPPS, and phosphate demonstrated a higher absorption at 470 nm (in the ascending order). This wavelength was selected to monitor the formation of insoluble Cu(II) aggregates (Figure 2) due to the absence of d–d absorption of initial Cu(II) complexes. Eventually, only Tris fully stabilized Cu(II) ions in solution, as indicated by the lack of turbidity and only a slight shift of the d–d band position and intensity during the 5 min period of observation (Figure S2). In PIPPS, the scattering of light on the formed Cu(OH)_2_ aggregates was so strong that the residual absorption band of the initial complex became barely discernible and could not be quantitated. In all other systems, the original absorption bands were clearly seen despite the baseline elevation due to light scattering on these aggregates. Comparing the d–d band parameters in the presence of phosphate with those of the Cu(II) aqua ion, the partially soluble Cu(OH)_2_ one can see that the Cu(II) ion is coordinated by phosphate rather than hydroxide ions. HEPES and MOPS systems did not evolve into pure Cu(OH)_2_ either but retained the coordinated buffer molecule. The formation of Cu(OH)_2_ particles at pH 6.0 in the MES system was only partial, leaving a significant portion of the Cu(H_2_O)_6_^2+^ ions in solution. The probably most unexpected result of this part of research is the highly differentiated evolution of systems containing HEPES, MOPS, and PIPPS buffers, despite their chemical similarity (Figure 1C). It shows the complicated nature of the observed precipitation processes. For four organic buffers, including MES, the effects of organic molecules on precipitate formation were strikingly differentiated. The PIPPS system evolved similarly to those of hydroxide and phosphate for about 10 s, whereas MES, HEPES, and MOPS systems underwent a rapid rearrangement process at ca. 60–70 s, followed by a more or less steady buildup of turbidity (Figure 2). They, however, evolved differently for the first minute of the process. For example, the Cu(II) ions in MES remained remarkably stable as apparent Cu(H_2_O)_6_^2+^ species for about a minute before the sudden increase of turbidity. The behavior of the MOPS system was the most peculiar one. Before the event at ca. 70 s, its turbidity evolved sigmoidally with a significant lag period, of ca. 2 s, during which the solution remained clear.

While the role of subtle differences in buffer molecule structures in Cu(OH)_2_ precipitation may pose an interesting research topic, deeper analysis of these phenomena is beyond the scope of this study. However, several important observations could be made as below.

1. All tested buffers suitable for pH 7.4 formed Cu(II) complexes within the dead time of the stopped-flow instrument.
2. The presence of phosphate, HEPES, or PIPPS buffers results in the formation of Cu(II) aggregates with no lag period, but with different aggregation rates. The aggregation in HEPES was significantly slower than in the other two buffers.
3. The MOPS buffer provided a remarkable time window (ca. 2 s under the tested conditions) in which no aggregates were visible. Therefore, it provides an option for studying fast reaction kinetics of Cu(II) ions at pH 7.4. One ought to remember, however, that the Cu(II) ion remains coordinated to the MOPS in this time window (see Table 1).
4. The analogous time window is as long as 60 s for MES at pH 6.0, with Cu(II) ions present as Cu(H_2_O)_6_^2+^. Hence, if the reaction can be studied at pH 6.0, then MES should be the buffer of choice.

### Effects of Buffers on the Interactions of Cu^2+^ Ions with GGH

**Figure 3:**
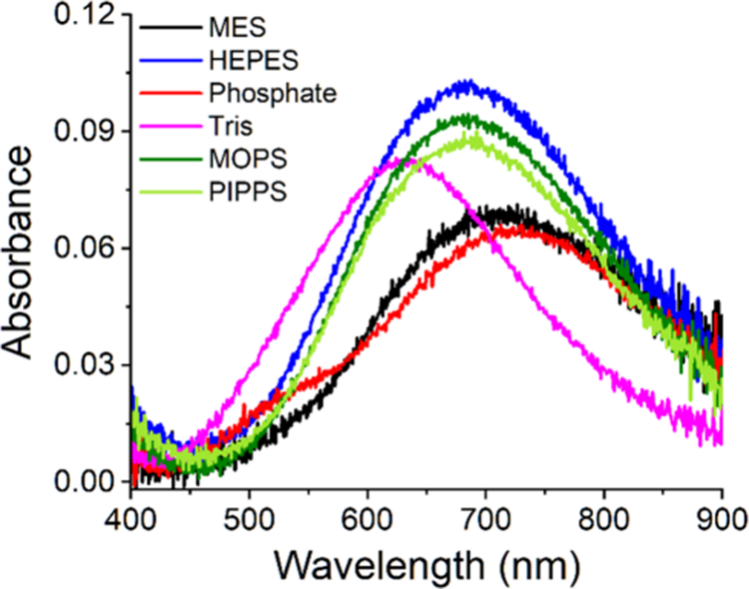
Initial spectra at 3.5 ms resulting from mixing of 3.2 mM Cu(H_2_O)_6_^2+^ ions (delivered by dissolving CuCl_2_ in distilled water) with the same volumes of 4 mM GGH dissolved in 400 mM buffers, pH 6.0 for MES and pH 7.4 for other buffers (final concentrations: 1.6 mM Cu(II) and 2 mM GGH in 200 mM buffer). The spectra are color-coded according to the labels in the graph.

We next studied how the buffers affect the Cu^2+^ reaction with GGH. All data are presented in Figures S4 and Figure 3 presents the initial spectra recorded at 3.5 ms of the reactions, while Figure S5 provides pairwise comparisons of the spectra recorded at 3.5 ms in the absence and presence of GGH. As shown in Figure 3, all buffers at pH 7.4 yielded spectra which differed from that of IC (the initial Cu(II)/GGH spectrum recorded in MES at pH 6.0;^19^ see Table 2 for parameters). The reaction in Tris started from the pure Tris complex, while in HEPES, MOPS, PIPPS, and phosphate, the initial spectra were different from both that of IC and those obtained in the absence of GGH. This demonstrates the initial formation of ternary complexes in all cases except Tris.

**Table 2:** Parameters and Assignments of d–d Bands Recorded at 3.5 ms for Reactions of Cu(H_2_O)_6_^2+^ Ions with GGH in Buffers at pH 7.4, Except for MES, pH 6.0. The relative error of λ_max_ determination is ±2 nm

buffer	λ_max_ (nm)	ε (M^–1^ cm^–1^)	coordination mode	refs
phosphate	724	40	2N [GGH] + O [HPO_4_^2–^]	this work
HEPES	684	64	2N [GGH] + N [HEPES]	this work
MOPS	683	58	2N [GGH] + N [MOPS]	this work
PIPPS	685	54	2N [GGH] + N [PIPPS]	this work
Tris	633	51	N + RO^–^/2N + RO^–^ [Tris]	(6)
MES (pH 6.0)	710	42	2N [GGH]	(19)

The initial d–d bands recorded in HEPES, MOPS, and PIPPS were blue-shifted by ca. 25 nm, and their intensities increased by ca. 50%, compared to MES (Table 2). This can be interpreted in terms of the reaction mechanism delineated previously.^19^ Due to the dead time of the stopped-flow system, the first species detected is IC (Scheme 1), a two-coordinate complex that contains a macrochelate loop between the amine and imidazole nitrogen atoms coordinated to Cu(II). This leaves two equatorial Cu(II) coordination sites available for the tertiary amine nitrogen of HEPES, MOPS, or PIPPS. As argued above, buffer molecules probably bind to Cu(II) much faster, in the single microsecond time scale, so the GGH rather binds to preformed Cu(II)-buffer complexes.

The d–d band blue shift observed for HEPES, MOPPS, and PIPPS is in accord with that, however, its extent is less than expected for a peptidic square-planar complex. It should be 50–70 nm rather than the observed 25 nm when quantified using the published formula of Sigel and Martin (see Table S1 in ref (19) for examples of such calculations).^27^ The less than expected blue shift could be caused by incomplete coordination, with the observed spectrum being a sum of contributions of 2N (IC) and 2N + N (ternary complex) chromophores. This is, however, less likely for 200 mM buffer concentrations and the estimated log *K* of 3–4 based on data for Cu(II) complexes of HEPES^4^ and MOPS.^7^ Alternatively, the diminished blue shift may suggest a lower than *D*_4*h*_ symmetry of the ternary complex,^28^ resulting from the huge sterical hindrance exerted by the bulk of tertiary amine (see Scheme 2). For the phosphate buffer, the initial d–d band was red-shifted by 14 nm vs IC. This effect is consistent with a replacement of a water molecule by phosphate in the IC coordination sphere.^29^

**Figure 4:**
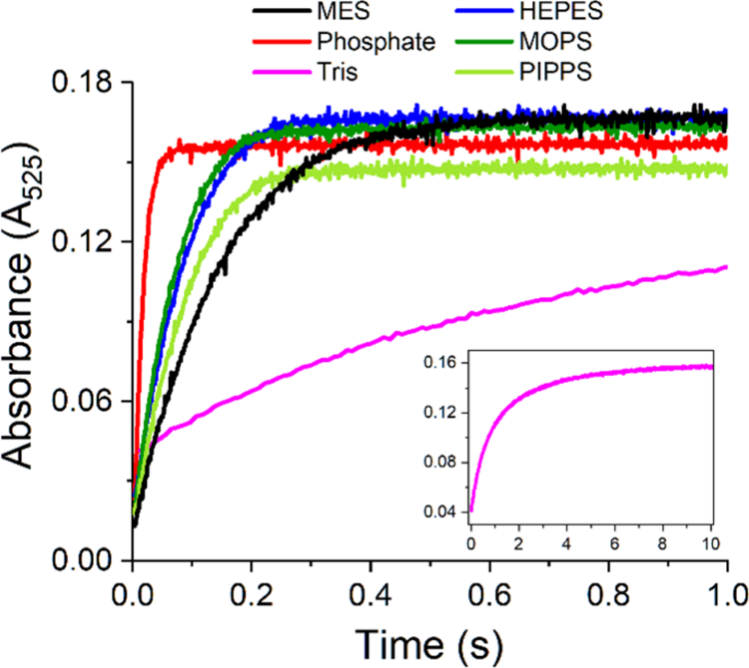
Absorbance changes at 525 nm corresponding to 4N CuGGH complex formation resulting from mixing of 3.2 mM Cu(H_2_O)_6_^2+^ ions (delivered by dissolving CuCl_2_ in distilled water) with the same volumes of 4 mM GGH dissolved in different 400 mM buffers, pH 6.0 for MES and pH 7.4 for other buffers (final concentrations: 1.6 mM Cu(II) and 2 mM GGH in 200 mM buffer). Inset presents the full course of the reaction in TRIS. The kinetic traces are color-coded according to the labels in the graph.

Table 3 provides rate constants calculated by monoexponential fitting of the reaction traces at 525 nm, as provided in Figure 4, with the monoexponential function. The experimental and fitted curves shown in Figure S6 were reasonably accurate for all buffers, except Tris, which could be more reliably fitted with a biexponential function. This deviation may stem from the presence of two Cu(II)/Tris complexes, CuTris and CuTris_2_ (see Table 2 and the related discussion). Nevertheless, all reactions exhibited isosbestic points, as shown in Figure S4. This means a single-step buffer molecule replacement by peptide nitrogens in the course of IC-to-4N conversion. Our previous studies performed in the absence of significant buffer interference indicated that the rate-limiting step of this conversion is the acquisition of a rare conformation by the GGH peptide chain. This conformation is suitable for simultaneous insertion of peptide nitrogens into the first coordination sphere of Cu(II).^19,22^ Reaction rates in HEPES, MOPS, and PIPPS were similar to each other, and somewhat faster than that in MES. This effect should be considered as apparent because the 4N complex formation is altogether strongly pH-dependent due to high intrinsic p*K*_a_ of peptide nitrogens,^15,27^ and the MES reaction was performed at pH 6.0 rather than 7.4. This issue is treated in more detail below.

**Table 3:** Observed Rate Constants (*k*_obs_) for the Formation of the 4N CuGGH Complex (Final Concentrations of 1.6 mM Cu(II), 2 mM GGH) in the Presence of 200 mM Buffers at pH 7.4 (Except for MES, pH 6.0)^a^

buffer	*k*_obs_ (s^–1^)	*t*_1/2_ (s)
phosphate	59.1(4)	0.012
HEPES	12.84(6)	0.054
MOPS	15.40(7)	0.045
PIPPS	12.69(7)	0.055
Tris	1.83(2) + 0.387(4)	0.72
MES (pH 6.0)	7.23(2)	0.096

a Statistical errors of determinations on the last significant digits are given in parentheses.

The presence of phosphate accelerated the 4N complex formation 5-fold, compared to HEPES, MOPS, and PIPPS, with ca. 5% of reaction product being formed already within the instrument dead time (a shoulder at 525 nm in the spectrum in Figure 3). In contrast, the reaction in Tris was more than 10-fold slower compared with other nitrogen buffers. At pH 7.4, Tris forms strong bis-complexes with Cu(II) ions, saturating its coordination sphere.^6^ Hence, one can propose that only the *mono*-complex, a minor species under the present conditions, is mechanistically susceptible to the GGH assault. This creates a bottleneck in the reaction of Tris with the Cu/GGH complex.

**Scheme 3:**
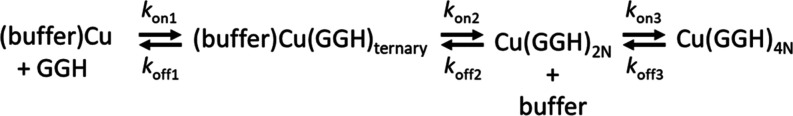
Steps of the Cu^2+^ Ion Reaction with GGH in the Presence of Coordinating Buffers, Based on Ref (19) and the Data Presented Above

Therefore, the order of events in buffered GGH reactions under conditions applied in this work is as follows: the Cu(II) ion is first intercepted by a buffer molecule, forming a 1:1 complex (except for Tris), which then reacts with GGH to form a ternary complex involving a peptidic macrochelate. Both these events take part within the dead time of the instrument but can be reconstructed on the basis of the published freeze-quench data for the Cu(II)/GGH system in MES.^19^ This ternary intermediate complex (TIC) undergoes a rate-limiting intramolecular reaction to yield the 4N species and simultaneous dissociation of the buffer molecule. The latter step is assured by the fact that the end point of all GGH reactions was identical with the 4N Cu(II)-GGH spectrum.^19^ The buffer competition step is not rate-limiting except for the Tris system, as discussed above. The *k*_obs_ obtained directly from the experimental data in this work corresponds to the ratio *k*_on3_/*k*_off3_ in Scheme 3.

**Figure 5:**
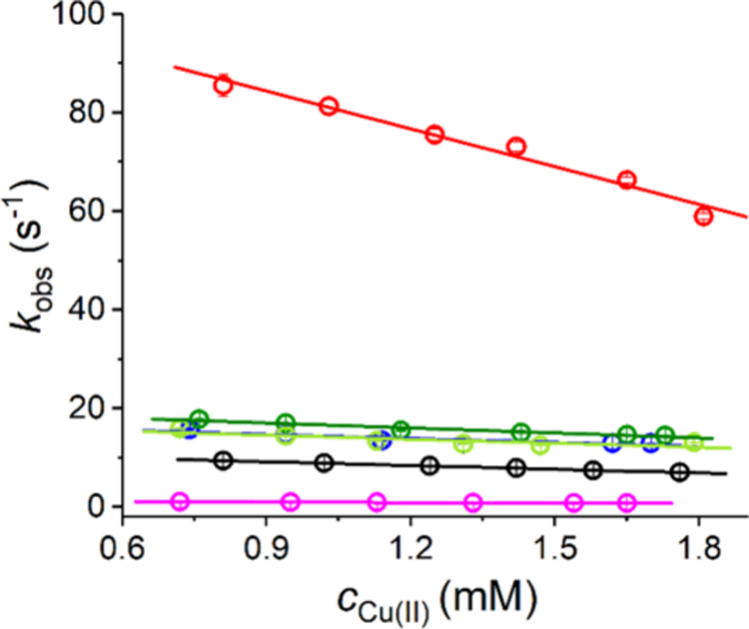
Dependences of observed rate constants (*k*_obs_) for the formation of 4N CuGGH complex on the *c*_Cu_/*c*_GGH_ ratio in the presence of different buffers: red, phosphate; black, MES; blue, HEPES; dark green, MOPS; light green, PIPPS; magenta, TRIS. *k*_obs_ values were calculated from the data obtained by mixing different copper concentrations (delivered by dissolving CuCl_2_ in distilled water) with the same volumes of 4 mM GGH dissolved in different 400 mM buffers (final concentrations: 0.7–1.8 mM Cu(II) and 2 mM GGH in 200 mM buffer). Error bars for individual determinations are within the symbols in all cases.

In order to gain deeper insight into these phenomena, the effects of 200 mM buffers on the Cu(II)/GGH reaction were studied over a broader range of Cu^2+^ to GGH ratios and pH values. The Cu(II) concentration effect was studied first, with GGH concentration fixed at 2 mM. The MES system in which there was little interference by the buffer in the Cu(II) reaction with GGH was also studied as a reference. In all instances, the Cu(II) concentration increase slowed the reaction, without affecting its one-step character. The linear dependence of *k*_obs_ on the Cu^2+^ to GGH ratio is presented in Figure 5, and parameters of the fit are given in Table S1.

We analyzed these data using the value of relative slope of straight line approximation of the reaction rate dependence on the GGH concentration (−B/A in Table S1). For MES at pH 6.0, the buffer molecule does not bind the Cu(II) ion effectively, and the reaction rate depends solely on intramolecular rearrangement of the coordinated GGH molecule, as presented in Scheme 1 and discussed in detail in ref. (22). The relative slope for this case was 0.22, which means that *k*_obs_ decreased by ca. 10% in the tested range of Cu(II) concentrations. The scale of this effect is thus not large. Remembering that the observed reaction step is intramolecular, and the formation of IC, its substrate, occurred before the beginning of the observation, one can consider free GGH to be a weak reaction catalyst. The contribution to this effect of the reaction product (the 4N complex) can be excluded because the exchange of Cu^2+^ ions between the 4N ATCUN/NTS complexes was studied previously and was found to occur on the time scale of days.^17,30−32^ Hence, we can narrow our considerations to the interactions of IC, which is labile.^19^ As demonstrated recently for a series of GGH analogues with individual nitrogen atoms modified by methylation or acetylation,^22^ the 4N complex formation, which involves the formation of two Cu–N bonds, is very highly cooperative. It requires a specific prearrangement of the peptide main chain (e.g., the concerted binding of two peptide nitrogens in unmodified GGH is faster than the formation of just a single one in either G(N–Me)GH or GG(N–Me)H). Taking this into account, we can propose that a larger excess of GGH molecules over IC allows for more efficient scanning for reactive peptide chain conformation by way of Cu^2+^ exchange among the GGH pool.

Quite interestingly, the apparent GGH catalysis also acts for Tris. The binding of Tris to the Cu(II) ion is so strong that it prevents the Cu(OH)_2_ precipitation in the absence of GGH (Figure 1). Accordingly, Tris slowed the 4N complex formation by an order of magnitude, but the GGH catalytic effect remained and was actually even slightly larger than for MES. A contribution of the GGH/Tris competition for Cu(II) binding could be responsible for this enhancement. Interestingly, the extent of the GGH catalytic effect is similar in the phosphate buffer, despite its opposite overall effect on the 4N formation rate (Table S1). The reasons are probably similar as well and involve GGH/phosphate competition.

The catalytic GGH effect was less pronounced for MOPS, HEPES, and PIPPS, with deviations from linearity. Molecules of these three buffers are structurally similar and are very bulky. Perhaps the coordinated buffer molecule slows down the postulated Cu^2+^ exchange between the GGH molecules, but, as clearly seen in Figure 5, this difference is small to negligible.

Phosphate buffer is a uniquely strong catalyst for the 4N complex formation. This effect is particularly relevant due to the role of phosphate as a component of physiological and laboratory buffers. Its ca. 10-fold acceleration of the studied reaction appears to reflect another phenomenon observed previously, namely, a nearly 4-fold reaction rate decrease by the amidation of the C-terminal carboxylate of GGH.^22^ The GGH carboxylate is not involved in Cu(II) binding in the 4N complex, as it points away from the coordinated Cu(II) ion.^33^ A steric hindrance is absent from the IC. The Cu(II)/carboxylate interaction can be further enabled in IC by the 2+ charge of the Cu(II) ion, but nevertheless, it rather has an indirect character. The reaction acceleration was observed in reactions of substituted GGH analogues containing the Glu residue in positions 2 or 4 (GEHG-amide and GGHE-amide, respectively), but the IC absorption spectra confirmed the absence of carboxylate coordination. In contrast, the carboxylate binding to the Cu(II) in the EGHG-amide IC complex slowed the reaction, rather than accelerating it.^22^ Therefore, an anionic group near the Cu(II) ion can accelerate the 4N complex formation if it does not impose limitations on the conformation of the Cu(II)-bound peptide.

**Scheme 4:**
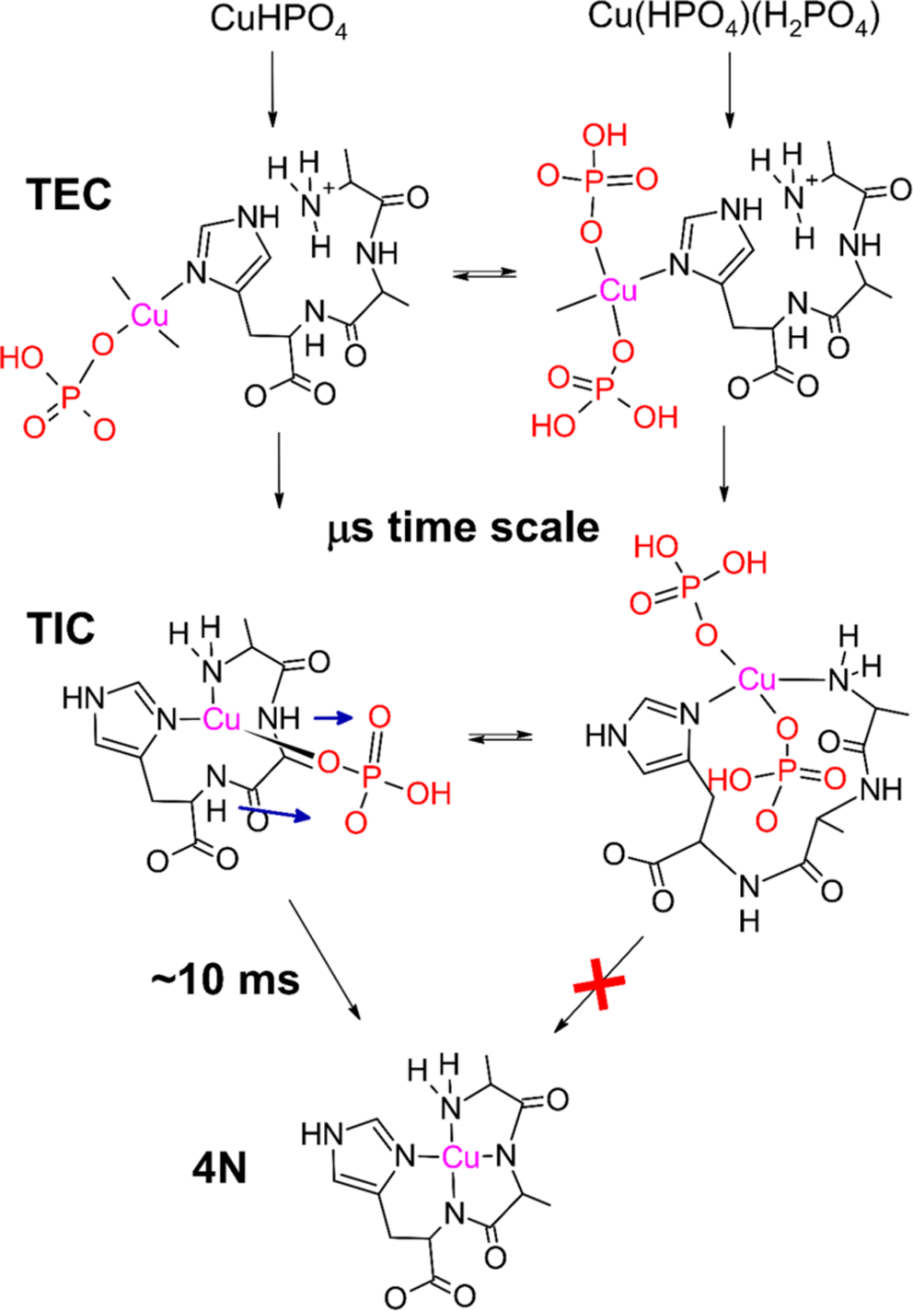
Hypothetic Mechanism of Catalysis and Inhibition of 4N Complex Formation in the Presence of a Phosphate Buffer The phosphate *mono-* and bis-complexes (CuHPO_4_ and Cu(HPO_4_)(H_2_PO_4_), the most abundant forms at pH 7.4 are shown) interact with GGH, initially forming ternary early complexes, TEC. These complexes undergo the first intramolecular rearrangement, yielding ternary intermediate complexes, TIC, which contain a macrochelate peptide loop. All of these processes are completed on the microsecond time scale under the given experimental conditions. In the TIC containing a single phosphate molecule, the GGH conformation productive in terms of the 4N complex formation is made available, and the 4N complex formation is enhanced by phosphate-assisted proton transfer, as indicated by blue arrows. In the TIC containing two phosphate molecules, such rearrangement is not possible until one coordinated phosphate ion dissociates from the Cu(II) ion. Charges and water molecules are omitted for clarity.

We tend to speculate that in the TIC, the bound phosphate can help withdraw the peptide bond proton upon the Cu(II) ion assault, thus accelerating the reaction (see Scheme 4 for a cartoon representation of this concept).

**Figure 6:**
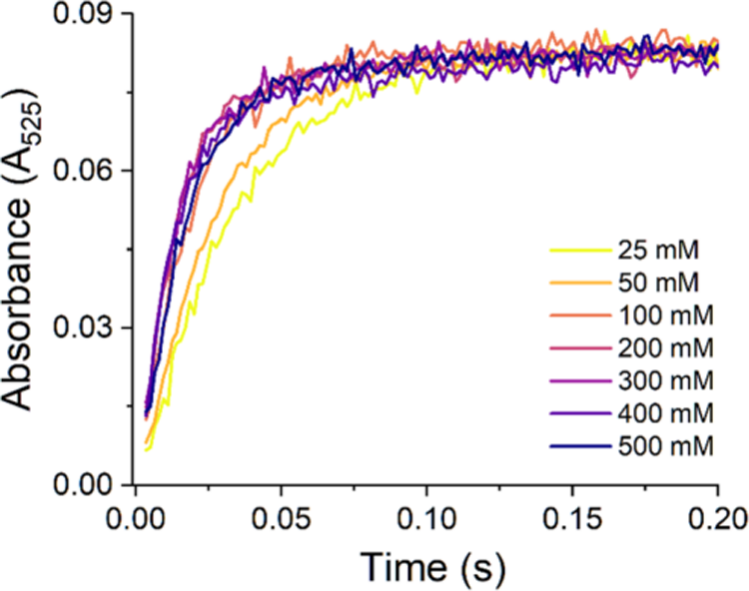
Absorbance changes at 525 nm corresponding to 4N CuGGH complex formation resulting from mixing of 1.6 mM Cu(H_2_O)_6_^2+^ ions (delivered by dissolving CuCl_2_ in distilled water) with the same volumes of 2 mM GGH in the presence of various phosphate concentrations, pH 7.4 (final concentrations: 0.8 mM Cu(II) and 1 mM GGH). The phosphate concentrations are color-coded according to the labels in the graph.

**Figure 7:**
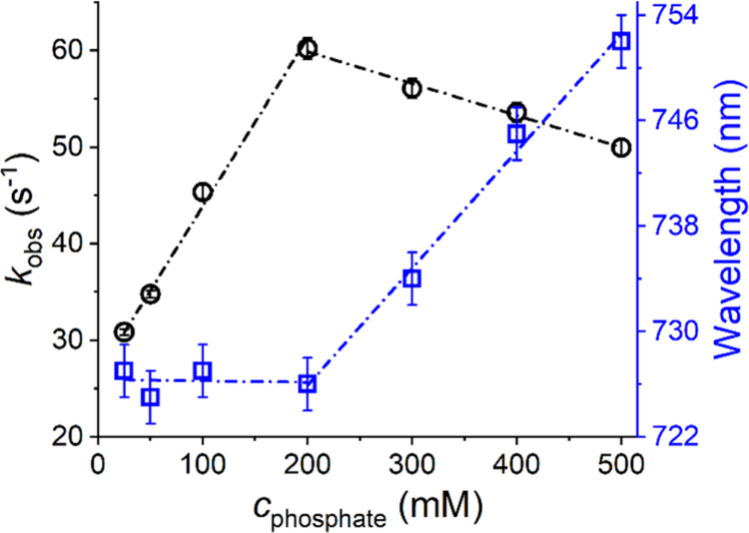
Dependences of calculated rate constants (black circles) and position of the d–d band of TIC at 3.5 ms (blue squares) for the formation of the 4N CuGGH complex at different phosphate concentrations (25–500 mM). Data points were determined from the absorbance spectra resulting from mixing of 1.6 mM Cu(H_2_O)_6_^2+^ ions (delivered by dissolving CuCl_2_ in distilled water) with the same volumes of 2 mM GGH in the presence of various phosphate concentrations, pH 7.4 (final concentrations: 0.8 mM Cu(II) and 1 mM GGH). Error bars for individual determinations are marked.

In order to better understand this phenomenon, we followed the effect of the phosphate concentration on the 4N complex formation. At all tested phosphate concentrations, the spectra evolved analogously to that presented in Figure S4, with clearly visible isosbestic points. The initial spectra of the TIC at 3.5 ms are presented in Figure S7. Figure 6 presents kinetic traces at 525 nm for these reactions, while monoexponential fits to these traces are presented in Figure S8. The obtained *k*_obs_ values are plotted in Figure 7 vs the phosphate concentration, and compared to the values of λ_max_ of TIC d–d bands. A very accurate correspondence between these parameters was found depending on the concentration range: In the range of 25–200 mM, the linear increase of *k*_obs_ was accompanied by a constant λ_max_ value of 726 ± 2 nm. Starting from 200 mM, the *k*_obs_ decreased linearly, which corresponded to a linear red shift of λ_max_, up to 753 ± 2 nm at 500 mM.

The excellent correspondence of these two parameters prompted us to provide the following explanation for the role of phosphate in the studied reaction. At concentrations up to 200 mM phosphate, the linear rate constant increase is due to catalysis by free, rather than coordinated phosphate, which can interact with both coordinated and free GGH molecules, enhancing the productive GGH conformation in both cases. The intercept of the line approximating this interaction, which extrapolates *k*_obs_ to zero phosphate, 27 ± 1 s^–1^, describes the contribution of the Cu(II)-bound phosphate ion to the reaction rate. It cannot be compared directly with the value for MES at pH 6.0 because the 4N formation process is pH-dependent on its own.^17^ The deceleration of the reaction above 200 mM phosphate can be then readily explained by the d–d band red shift, which must be caused by the binding of another phosphate ion to TIC. The weak binding constant about 1 M^–1^, expected for this interaction, explains the linearity of the effect in the studied pH range. The resulting reaction inhibition mechanism is analogous to the case of Tris because electrostatics forces the two phosphate ions to *trans* positions around the Cu(II) ion. This would preclude productive GGH coordination. The structural aspects of these interactions are also illustrated in Scheme 4.

**Figure 8:**
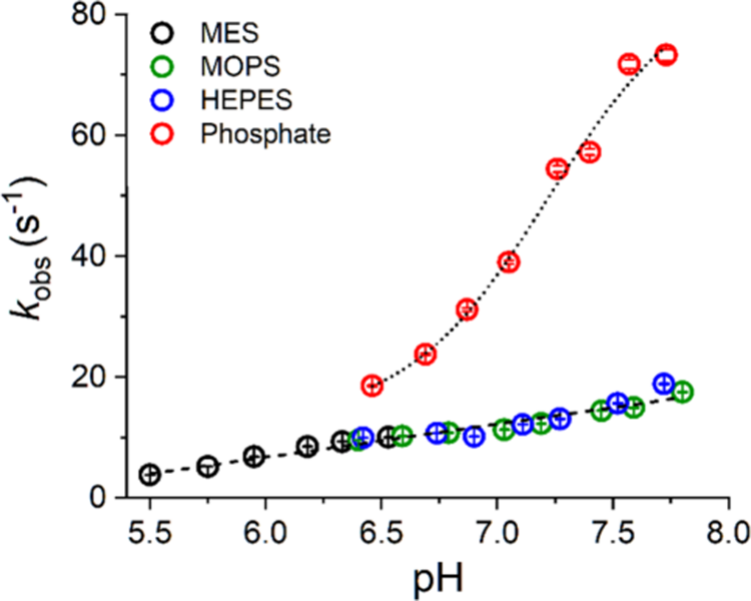
pH dependence of *k*_obs_ determined for the reaction of 4N CuGGH complex formation in different buffers. The dotted line represents the sigmoidal fit (Hill equation, p*K* 7.22 ± 0.09, *n* = 1.4 ± 0.3, *R*^2^ = 0.995) to phosphate data, and the dashed line represents the linear fit to MES, MOPS, and HEPES data taken together (*y* = *A* + *B* × *x*; *A* (intercept) = −26 ± 1 s^–1^, *B* (slope) = 5.5 ± 0.2 s^–1^/pH unit; *R*^2^ = 0.97). Circles representing data points are color-coded according to the labels in the graph. Error bars for individual determinations are within the symbols in all cases.

Further insight into the role of buffers in the Cu(II)/GGH reaction was provided by experiments at various pH values. These experiments were performed for 200 mM MES, phosphate, HEPES, and MOPS buffers in the pH ranges of 5.5–6.5, 6.5–7.7, 6.4–7.7, and 6.4–7.8, respectively. The results are summarized in Figure 8. The pH dependence of *k*_obs_ in phosphate buffer had a sigmoidal character and could be fitted with the Hill equation.^34^ The obtained p*K* value was 7.22 ± 0.09, with the Hill (cooperativity) coefficient of *n* = 1.4 ± 0.3. This apparent p*K* is higher than that of the H_2_PO_4_^–^ ion dissociation by 0.5 pH units, and the value of *n* for the latter process should be 1. Therefore, the observed effect must originate from a more complicated interaction, which corroborates the structural effect of phosphate ions on GGH, as postulated in the preceding section. One should note, however, that the acceleration of the studied reaction with increasing pH is primarily due to the enhancement of Cu(II)-assisted deprotonation of GGH peptide nitrogen atoms, which is its underlying driving force. This is reflected in the overall trend seen for MES, HEPES, and MOPS reactions, which could be approximated quite well with a linear function. The linearized trends for individual buffers are similar to the overall trend. Small deviations seen in Figure 8 are due to specific interactions of these buffers in the reactive TIC, but, very clearly, they contribute only a little to the reaction rate, as presented above. The estimated p*K* value for spontaneous (Cu(II)-independent) deprotonation of GGH peptide nitrogens is 15 or higher, which explains the linearity of the overall effect in the studied pH range.^27^

The key results of the study of 4N complex formation in the presence of buffers are listed below.

1. All buffers listed by Ferreira et al.^8^ as “noncoordinating” in fact interfered with the 4N complex formation at pH 7.4 because, as evidenced by initial absorption spectra, they formed Cu(II) complexes prior to the observation window of stopped-flow experiments.
2. In all cases, the reaction outcome and overall mechanism remained unaffected by the buffer. The effect was limited to the reaction rate, and hence buffers can be interpreted as catalysts or inhibitors of the 4N complex formation.
3. The effect of morpholine and piperazine buffers, MES, MOPS, HEPES, and PIPPS on the rate of 4N complex formation reaction was small and can be considered as perturbation to the general linear dependence of the rate constant *k*_obs_ on pH.
4. Tris was a strong reaction inhibitor due to efficient Cu(II) chelation.
5. In contrast, phosphate was a strong reaction catalyst. This finding is particularly important as phosphate is the component of physiological buffers.

## Conclusions

We investigated how various common buffers affect the rate of reaction of the binding of Cu^2+^ to the GGH peptide. We also studied the background reactions of Cu^2+^ ions with buffers alone. All buffers formed Cu(II) complexes within the 3.5 ms delay between the sample mixing and the spectral recording, both without and with GGH. As expected, Cu(OH)_2_ precipitates were eventually formed for all buffers except Tris. However, significant time windows were found for morpholine buffers MES and MOPS between the reaction onset and the formation of the Cu(OH)_2_ precipitate. This feature may be useful in designing future experiments. The presence of buffers did not affect the final reaction product, the 4N Cu/GGH complex, but affected the reaction rates. The effect was small in morpholine and piperazine buffers. Two other studied buffers exhibited much stronger interference. Tris was a strong reaction inhibitor due to effective Cu(II) complexation. In contrast, phosphate accelerated the reaction rate. The results of our study can be considered as guidelines for planning and interpreting kinetic experiments involving Cu(II) ions and small biomolecules.

## Supporting Information Available

Full spectra recorded in stopped-flow studies of Cu^2+^ ion interactions with buffers in observation periods of 1.5 s and 5 min, respectively (Figures S1 and S2); absorption maxima of spectra provided in Figure S1 are tracked and these figures are auxiliary for Figure 1 (Figure S3); full spectra of Cu^2+^ ion binding to GGH in the presence of buffers at pH 7.4 (except MES, pH 6.0) and this figure is auxiliary to Figures 3 and 4 (Figure S4); comparison of initial kinetic spectra recorded in the absence and presence of GGH (Figure S5); monoexponential fits to kinetic traces plotted in Figure 4 (Figure S6); initial spectra recorded for Cu^2+^ reactions with GGH at various concentrations of phosphate (Figure S7); monoexponential fits to kinetic traces plotted in Figure 6 (Figure S8); and coefficients for linear fits for the data presented in Figure 5 (Table S1) (PDF)
