# Supplementary material for: Nobody’s Perfect: Choice of the Buffer and the Rate of Cu2+ Ion–Peptide Interaction
Source: Inorg Chem. 2024 Jun 14;63(26):12323–32. doi: 10.1021/acs.inorgchem.4c01797 (PMC11220749; doi:10.1021/acs.inorgchem.4c01797)
Supplement: Supplementary file 1 — ic4c01797_si_001.pdf [file ic4c01797_si_001.pdf]

Supporting Information for

Nobody's Perfect: Choice of the Buffer and the Rate of Cu^{2+} Ion–Peptide Interaction

Radosław Kotuniak, Dobromiła Z. Sudzik, Iwona M. Ufnalska,* and Wojciech Bal*

Institute of Biochemistry and Biophysics, Polish Academy of Sciences, Pawińskiego 5a, 02-106 Warsaw, Poland

* Corresponding authors. E-mail iufnalska@ibb.waw.pl, wbal@ibb.waw.pl

Phone: +48-22-592-2370

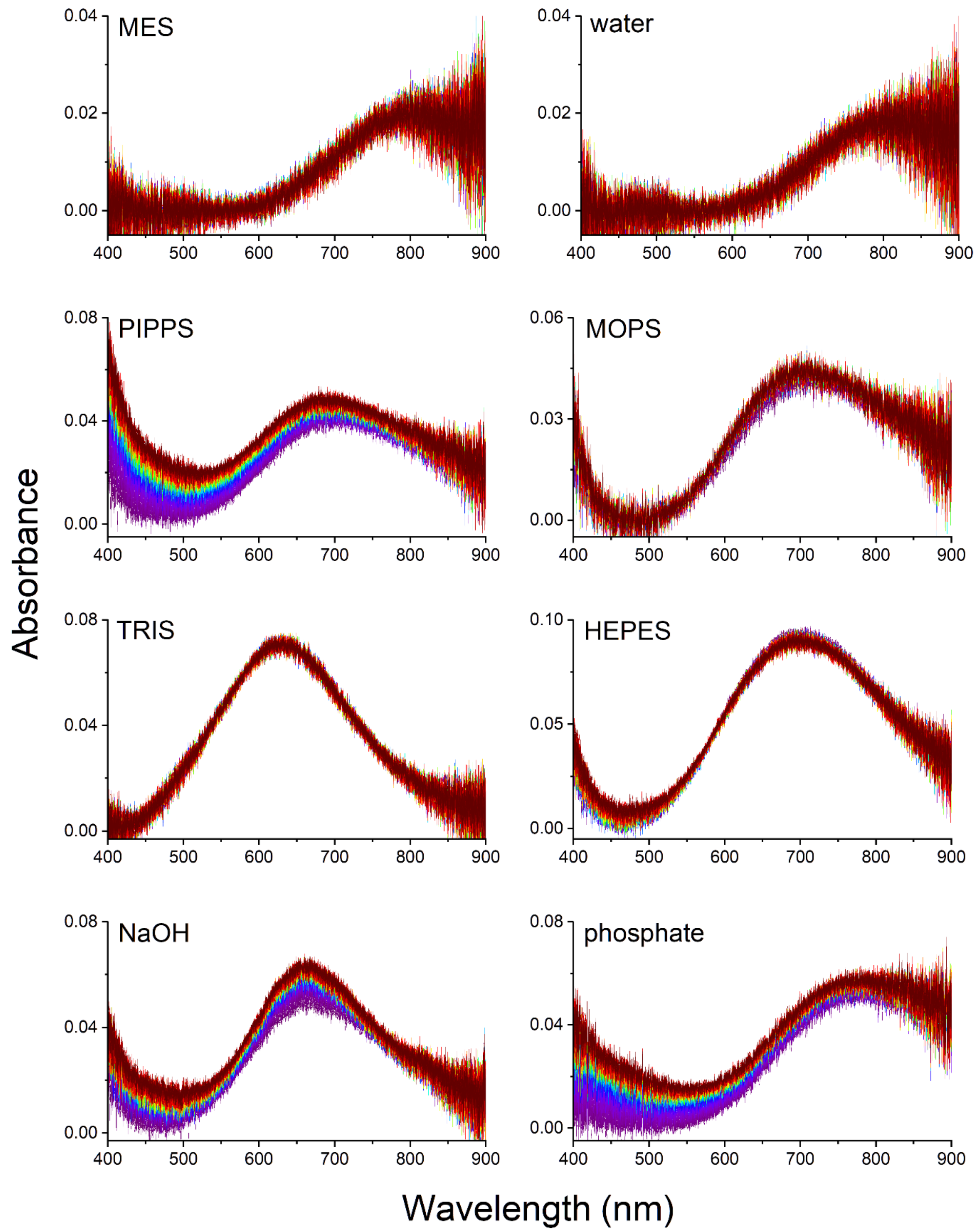

Figure S1. The absorption spectra collected for 1.5 s after mixing of 3.2 mM $Cu(H_2O)_6^{2+}$ ions (delivered by dissolving $CuCl_2$ in distilled water) with the same volumes of 400 mM buffers, pH 6.0 for MES and pH 7.4 for other buffers (final concentrations: 1.6 mM Cu(II) and 200 mM buffer). Additional reactions of 3.2 mM $Cu(H_2O)_6^{2+}$ ions with water and with 50 mM NaOH (final pH 12.4) were performed. The labels in the individual graphs indicate buffer/solution used in the experiment.

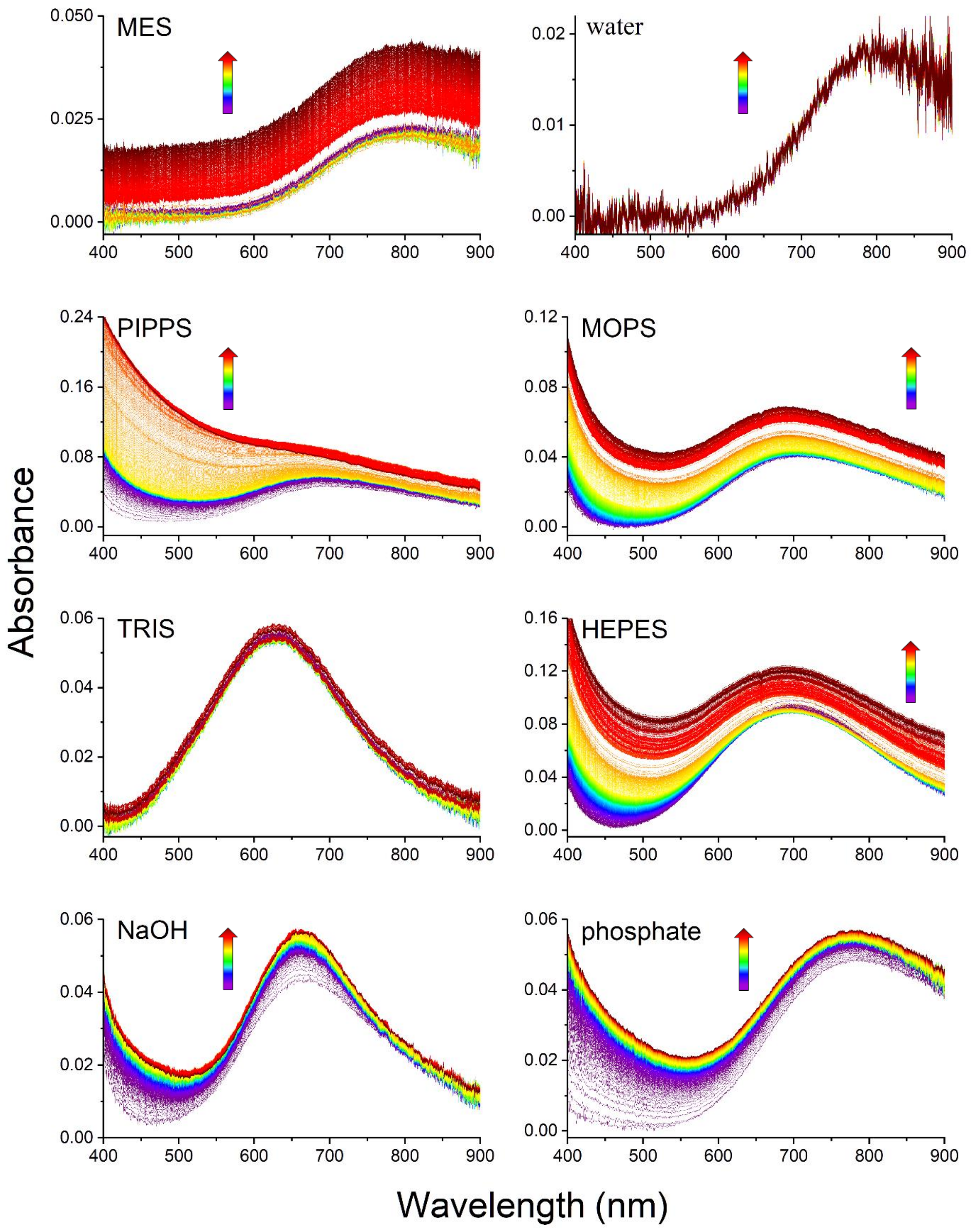

Figure S2. The absorption spectra collected for 5 min after mixing of 3.2 mM $Cu(H_2O)_6^{2+}$ ions (delivered by dissolving $CuCl_2$ in distilled water) with the same volumes of 400 mM buffers, pH 6.0 for MES and pH 7.4 for other buffers (final concentrations: 1.6 mM Cu(II) and 200 mM buffer). Additional reactions of 3.2 mM $Cu(H_2O)_6^{2+}$ ions with water and with 50 mM NaOH (final pH 12.4) were performed. The spectra are color coded according to colors of arrows shown in the graphs. The labels in the individual graphs indicate buffer/solution used in the experiment.

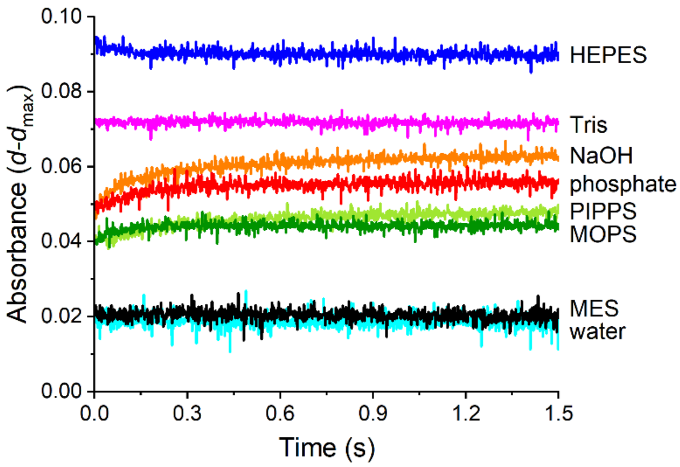

Figure S3. Absorbance changes at initial peak maxima (given in Table 1) resulting from mixing of 3.2 mM $Cu(H_2O)_6^{2+}$ ions (delivered by dissolving $CuCl_2$ in distilled water) with the same volumes of 400 mM buffers, pH 6.0 for MES and pH 7.4 for other buffers (final concentrations: 1.6 mM Cu(II) and 200 mM buffer). Additional reactions for 3.2 mM $Cu(H_2O)_6^{2+}$ ions with water and with 50 mM NaOH (final pH 12.4) were performed. Kinetic traces are color coded according to the labels in Figure 3.

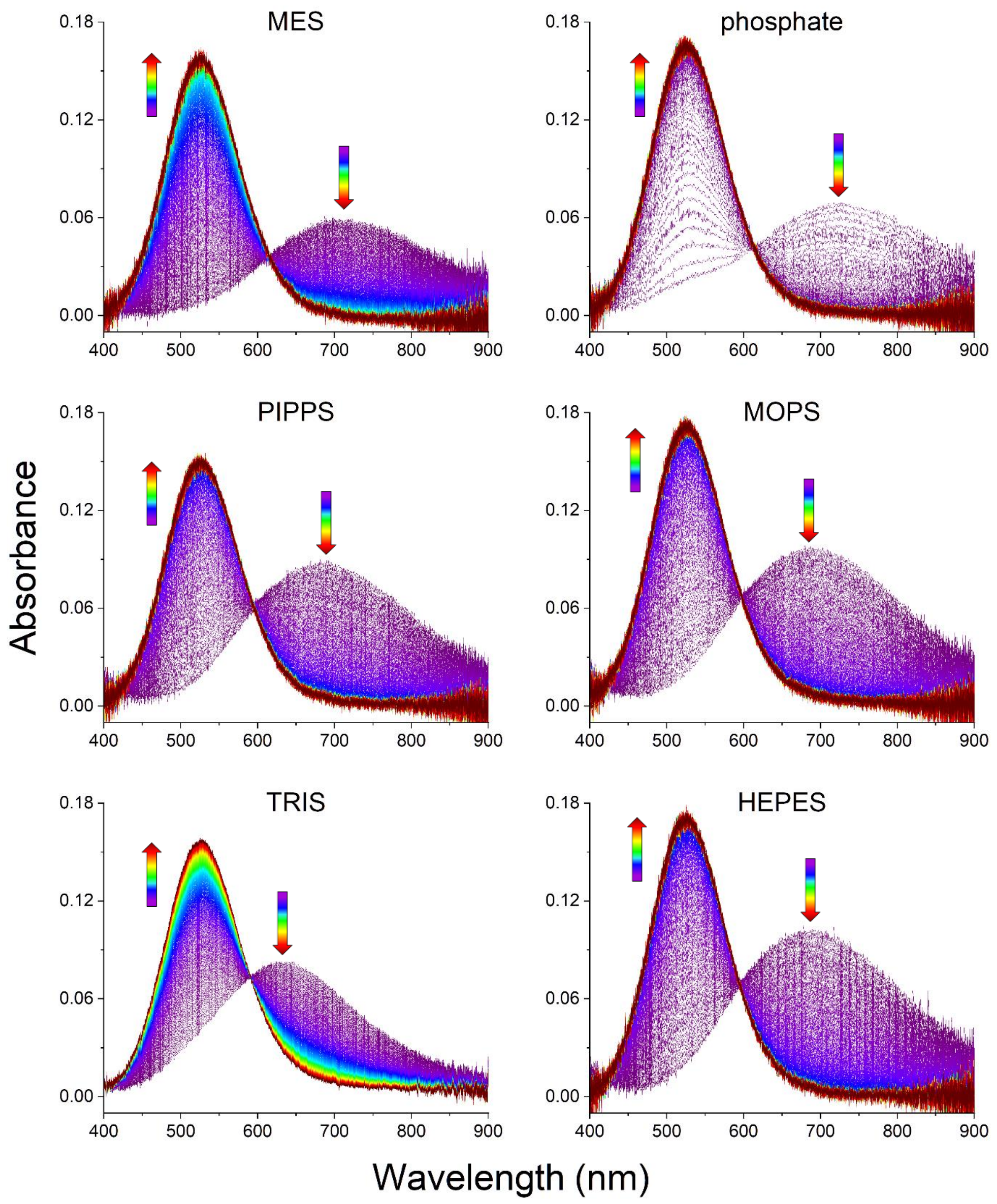

Figure S4. The absorption spectra collected for 1.5 s after mixing of 3.2 mM $Cu(H_2O)_6^{2+}$ ions (delivered by dissolving $CuCl_2$ in distilled water) with the same volumes of 4 mM GGH in 400 mM buffers, pH 6.0 for MES and pH 7.4 for other buffers (final concentrations: 1.6 mM Cu(II) and 2 mM GGH in 200 mM buffer). An exception is TRIS for which data were collected for 10 s. The labels in the individual graphs indicate buffer used in the experiment. The spectra are color coded according to colors of arrows shown in the graphs. Isosbestic points were observed for all reactions, at the following wavelengths: 612 nm for phosphate, 594 nm for HEPES , 595 nm for PIPPS and MOPS, 590 nm for Tris and 615 nm for MES.

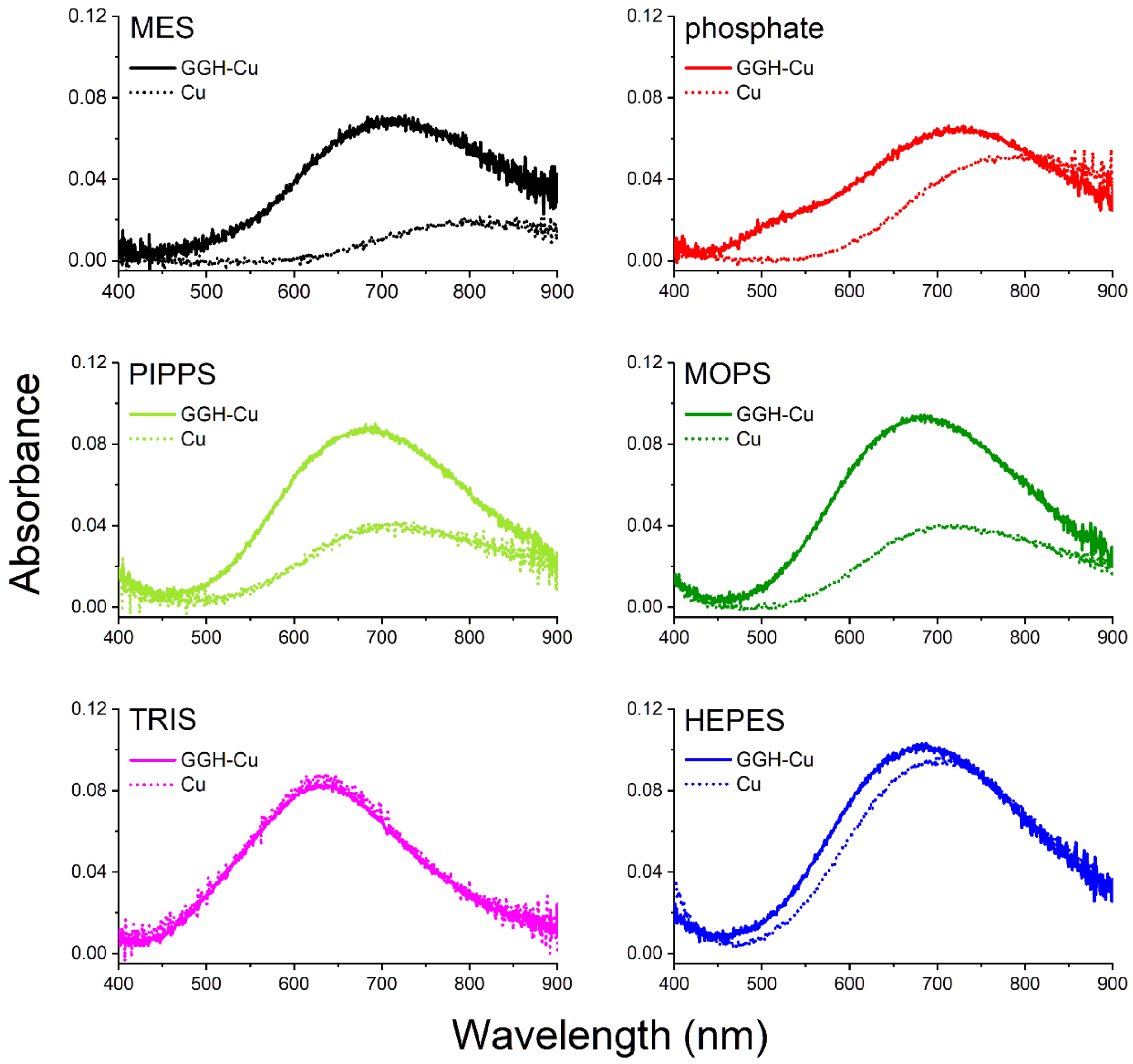

Figure S5. A comparison of initial *d-d* spectra at 3.5 ms resulting from mixing of 3.2 mM $Cu(H_2O)_6^{2+}$ ions (delivered by dissolving $CuCl_2$ in distilled water) with the same volumes of 400 mM buffers in the absence (dotted lines) and presence of 4 mM GGH (pH 6.0 for MES, pH 7.4 for the rest of buffers; final concentrations: 1.6 mM Cu(II), 2 mM GGH and 200 mM buffer). The labels in the individual graphs indicate buffer used in the experiment.

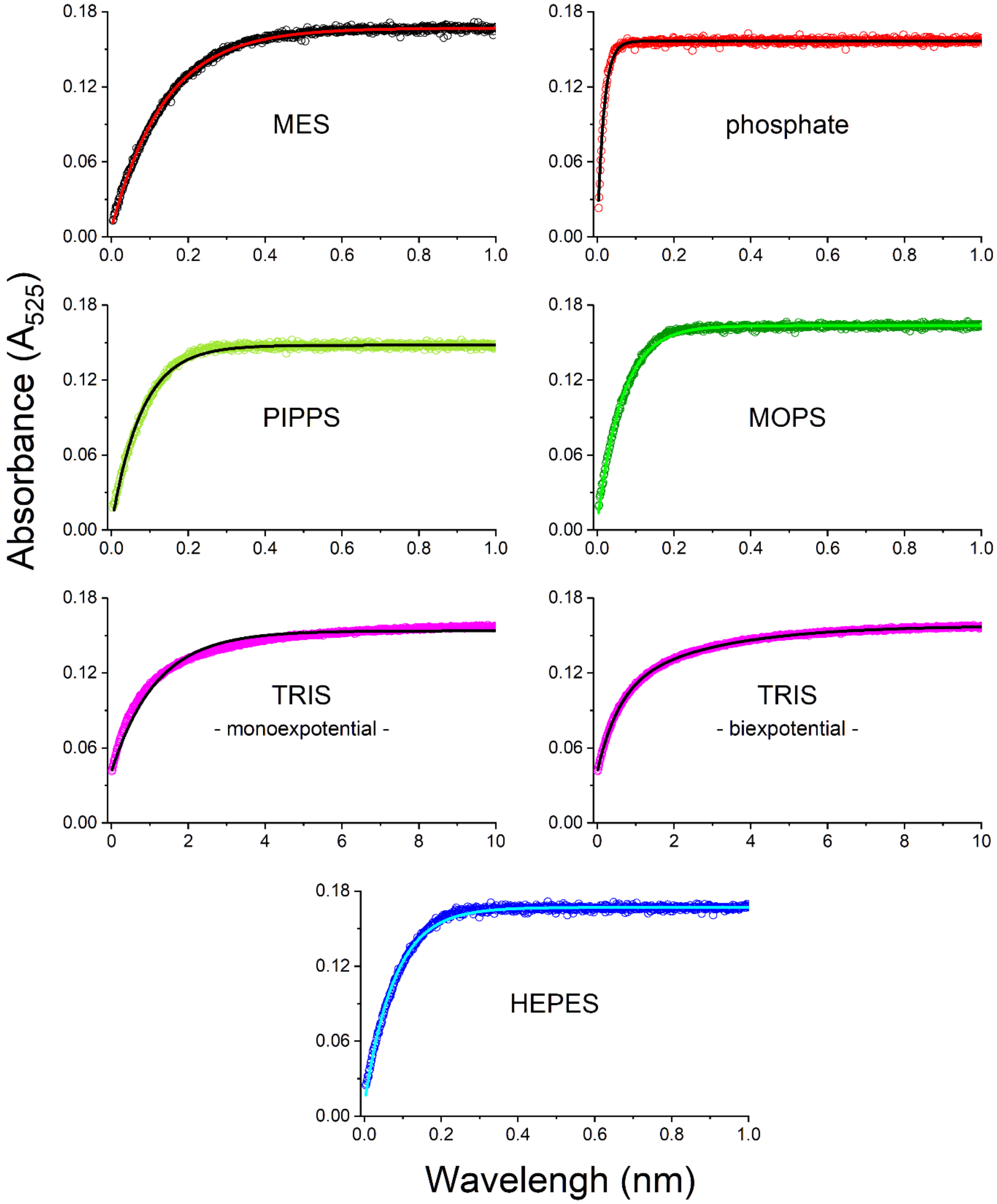

Figure S6. Monoexponential fits to kinetic traces plotted in Figure 4, corresponding to 4N CuGGH complex formation resulting from mixing of 3.2 mM $Cu(H_2O)_6^{2+}$ ions (delivered by dissolving $CuCl_2$ in distilled water) with the same volumes of 4 mM GGH dissolved in different 400 mM buffers, pH 6.0 for MES and pH 7.4 for other buffers (final concentrations: 1.6 mM Cu(II) and 2 mM GGH in 200 mM buffers). In case of TRIS biexpotential fit was also performed.

Table S1. Linear fit coefficients and quality of fit parameter (R^2) for the dependence of rate of formation of the 4N CuGGH complex on Cu(II)/GGH ratio in the presence of 200 mM buffers, pH 7.4, except of MES, pH 6.0. The GGH concentration was fixed at 2 mM. Statistical errors of determinations on the last significant digits are given in parentheses.

Buffer	*k* = A + B × ([Cu(II)]/[GGH])			Relative line slope (–B/A)
	A	B	R^2	
Phosphate	107(3)	-26(2)	0.976	0.24
Tris	1.24(5)	-0.32(4)	0.946	0.26
MES (pH 6.0)	11.40(9)	-2.51(6)	0.997	0.22
HEPES	17(1)	-2.8(8)	0.772	0.16
MOPS	20.0(5)	-3.3(4)	0.944	0.17
PIPPS	17(1)	-2.7(9)	0.681	0.16

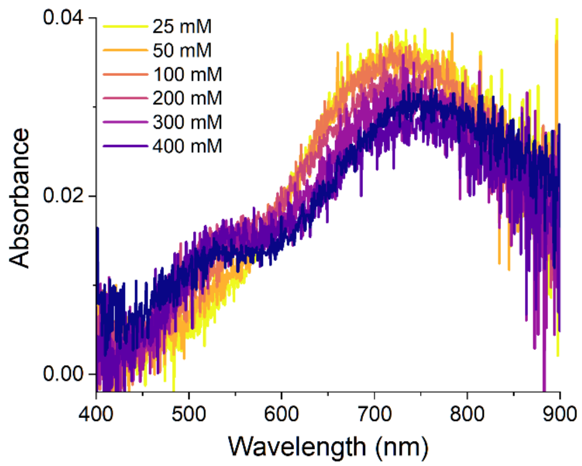

Figure S7. The initial spectra at 3.5 ms resulting from mixing of 1.6 mM $Cu(H_2O)_6^{2+}$ ions (delivered by dissolving $CuCl_2$ in distilled water) with the same volumes of 2 mM GGH in the presence of various phosphate concentrations, pH 7.4 (final concentrations: 0.8 mM Cu(II) and 1 mM GGH). The phosphate concentrations are color coded according to the labels in the graph.

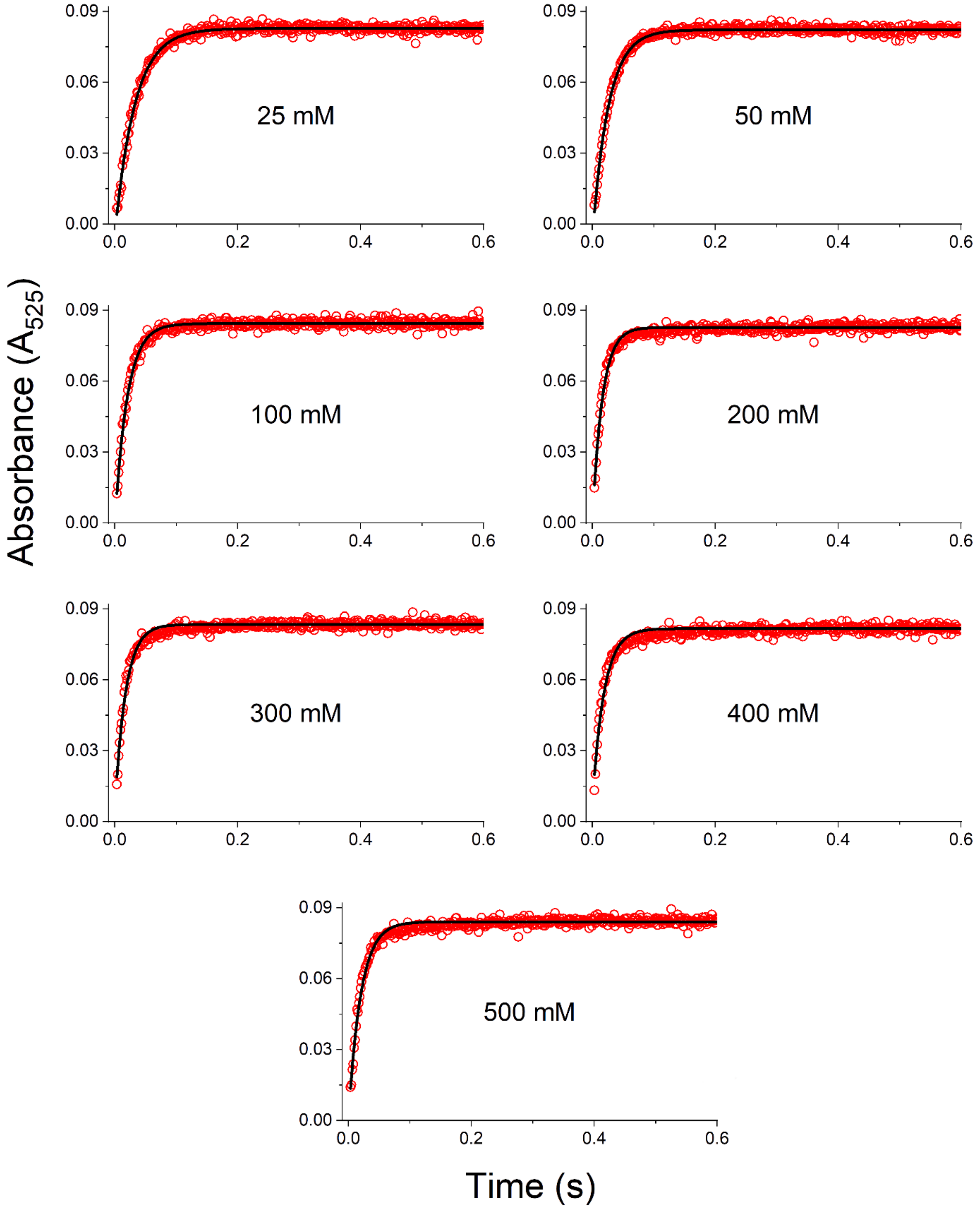

Figure S8. Absorbance changes at 525 nm corresponding to 4N CuGGH complex formation resulting from mixing of 1.6 mM $Cu(H_2O)_6^{2+}$ ions (delivered by dissolving $CuCl_2$ in distilled water) with the same volumes of 2 mM GGH in the presence of various phosphate concentrations, pH 7.4 (final concentrations: 0.8 mM Cu(II) and 1 mM GGH). The labels in the individual graphs indicate final phosphate concentrations in each experiment.
